# Evaluation of the biocontrol activity of *Frateuria defendens*-derived metabolites against mollicutes

**DOI:** 10.1080/15592324.2022.2070355

**Published:** 2022-04-29

**Authors:** Alaa Naama-Amar, Yoram Gerchman, Lilach Iasur Kruh, Vered Naor

**Affiliations:** a Department of Biotechnsology Engineering, ORT Braude College of Engineering, Karmiel, Israel; b Department of Evolutionary and Environmental Biology, Haifa University, Haifa, Israel; c Golan Agri Innovation Unit, Shamir Research Institute, Katsrin, Israel

**Keywords:** bacterial biocontrol agents, Frateuria defendens, Mollicutes, Small Bioreactor Platform

## Abstract

*Frateuria defendens* is a candidate biocontrol agent that has been shown to reduce phytoplasma-related disease symptoms in grapevines and periwinkle plants. While a crude filtrate prepared from *F. defendens* can inhibit mollicute growth, the specific growth parameters for this bacterium, necessary to enhance this protective inhibitory response, remain unknown. Moreover, the separation of filtrate preparations from bacterial cells via centrifugation and filtration is laborious and time-consuming. As such, the present study was conducted to define the optimal growth conditions associated with maximal inhibitory activity of *F. defendens* and to establish a better approach to separating these bacterial cells from their secreted metabolites. To conduct these analyses, *F. defendens* was cultured in a range of media types, while associated inhibitory effects were tested *in vitro* using *Spiroplasma melliferum* as a model mollicute bacterium, and *in planta* using phytoplasma-infected periwinkle plantlets. These analyses revealed *F. defendens* growth patterns change based upon media composition, with filtrates prepared from a specific rich medium (S-medium) exhibiting beneficial activities, including the inhibition of *S. melliferum* and enhanced plant growth. When *F. defendens* cells were grown within semi-permeable, membrane-coated Small Bioreactor Platform (SBP) capsules, they could be more readily separated from the secreted metabolite fraction, obviating the need for filtration and/or centrifugation. This study is the first to have reported the use of SBP capsules to separate bacterial cells from their secreted metabolites under sterile conditions while retaining the ability of these metabolites to inhibit *S. melliferum* growth and to benefit the host plant. The results highlight promising new approaches to the effective biocontrol of phytoplasma-driven diseases in grapevines and other economically important plant species.

## Introduction

Yellows disease in grapevine causes substantial damage to this high-value crop and yet remains difficult to control. The causative agents of yellows-type diseases are a range of phloem-inhabiting phytopathogenic mollicute bacteria, referred to as phytoplasma, that are vectored by insects of the Hemiptera order.^1^ Bois noir, which is caused by *Candidatus* Phytoplasma solani, is the only form of yellows disease known to affect grapevines in Israeli vineyards.^2^ Efforts to control this disease via uprooting infected grapevines or deploying insecticides against the planthopper *Hyalsthes obsoletus*, which is known to vector this disease, have proven ineffective methods of disease control. Recently, the use of bacterial biocontrol agents (bBCA) to control phloem-inhabiting phytopathogen was suggested.^3^ In general, bBCA inhibit plant disease through three possible pathways: (1) Competing with phytopathogens for space and nutrients; (2) Secreting antibiotic substances that inhibit plant pathogens; (3) Activating the plant’s immune system via the mechanism of induced systemic resistance (ISR).^4^

*Frateuria defendens* is a novel endophytic bacterium isolated from *H. obsoletus*.^5^ *F. defendens* has been shown to penetrate plants via roots or leaf stomata, whereupon it can inhabit the xylem and phloem of grapevines and many other plants without apparent phytotoxicity.^6^ *F. defendens* application has been reported to reduce the symptoms of phytoplasma-induced yellows disease in potted grapevine and periwinkle plants, and in grapevines under field conditions, suggesting the potential for the application of *F. defendens* as a bBCA.^7–10^ Information about this beneficial bacterium and its possible mode of action was obtained by different methods in previous studies: (1) Microscopic analyses have shown that *F. defendens* settle in the plant phloem, therefore suggesting that it competes with phytoplasma for the same niche;^7^ (2) Genomic analyses did not detect genes that are correlated with the plant ISR;^8^ (3) Chemical analyses have shown that *F. defendens* secrete several substances that inhibit the growth of Mollicutes bacteria.^9^ Hence, we hypothesize that the secreted metabolites of *F. defendens*, combined with its location, are the main mechanism that results in the reduction of disease symptoms in phytoplasma-infected plants. However, the inhibitory effects of *F. defendens* on infected grapevines and its survival period under field conditions are lower than those observed during laboratory testing.^10^ The utilization of *F. defendens-*derived secreted metabolites has thus been proposed as a potential means of overcoming this problem, with *F. defendens* filtrates having been shown to significantly inhibit the growth of the cultivable Mollicutes *Spiroplasma melliferum* (which is used as a surrogate for phytoplasma) under laboratory conditions.^5^ Furthermore, applying *F. defendens* filtrate *in planta* reduced the mortality rate of periwinkle plantlets infected by yellows disease,^9^ affirming the potential utility of the filtrate as a tool for biological control.

Production and collection of filtrate (extra-cellular microbial metabolites) can be achieved in many ways. The most common approach is to grow the microbial cells in a broth until the desired growth stage, followed by separating the cells from the broth by filtration or centrifugation in order to obtain a clear broth from which the desired metabolites can be extracted.^11^ One approach to enable this separation is the immobilized cells methodology.^12–15^ This approach was proven to be effective in the utilization of microbial enzyme production^12^ and other substances, such as balsamic vinegar^13^ and ethanol.^14^ Direct extraction of the metabolites from the broth is another approach. This can be accomplished through crystallization (e.g., after pH adjustment^16^ liquid-liquid extraction,^17^ nanofiltration,^18^ forward osmosis, or by using solid absorbents such as silica gel or alumina.^19^

Indeed, the efforts to separate and identify the inhibitory substances present within *F. defendens* filtrate, using solid absorbents combined with extraction with different solvents, have led to the identification of 4-quinolincarboxyaldehyde and 5-hydroxymethylfuraldehyde within the filtrate active fraction.^9^ However, these compounds exhibited lower inhibitory activity compared to the crude filtrate, suggesting that other compounds are involved in the inhibitory process.^9^ Indeed, bacteria-derived filtrates often contain a wide variety of compounds with antibiotic activity, as in the case of *Trichoderma* spp., which secrete ca. 100 antibiotic substances.^20^ Moreover, filtrates prepared from *Pseudomonas fluorescens* FF48 cultures have been reported to inhibit *Flavobacterium psychrophilum* biofilm formation,^21^ and *Bacillus subtilis* CW14 culture-derived filtrates inhibited *Aspergillus ochraceus* growth.^22^ In some instances, the use of bacteria-free filtrate has been reported to be more effective than applying the bBCA cells.^23^ For example, *Pseudomonas fluorescence* AS15 exhibits a lower inhibition rate (35.5%) against *Rhizoctonia solani*, the causal pathogen of banded leaf and sheath blight disease, when grown under a dual plate assay, compared to that observed when applying the cell-free filtrate (86.6%).^24,25^ As such, bacteria-derived filtrates are complex, rich solutions, with the potential to inhibit microbial growth more effectively than specific purified compounds or living bacteria cells. Nevertheless, effective and reliable approaches are required for the preparation and purification of such filtrates.

In an effort to build upon the above observations, the present study was developed with the goal of optimizing the growth conditions for *F. defendens* in order to maximize its inhibitory activity against *S. melliferum* and to establish the most effective approach to separating *F. defendens* cells from metabolite-rich filtrates produced therefrom. The results of these analyses highlight promising new biological approaches to the control of phytoplasma diseases in grapevines and other economically important plant species.

## Materials and methods

### Bacterial strains

*S. melliferum* and *F. defendens* culture stocks from the laboratories of Zchori-Fein (Newe Yaar, Agricultural Research Organization, Israel) and Naor (Shamir Research Institute, Israel) were used for all experiments.^5^ Both of these bacteria were routinely grown in S-medium (see below), and were stored in 100–200 μl aliquots in 50% glycerol at −80^°^C. Starter cultures were prepared by adding one aliquot to 15 ml of medium, and cells were grown at 28^°^C to log phase (5 days for *S. melliferum*; 2 days for *F. defendens*), reaching approximately 3 × 10^7^ CFU/mL.

### *Phenotypic characterization of* F. defendens

The 96-well panelGEN MicroPlate (Biolog, USA) was used for *F. defendens* characterization. This assay utilized a 96-well plate in which each well contained a different carbon source, distinct growth conditions (pH/ salinity), or inhibitors, together with tetrazolium redox dyes. *F. defendens* growth in a given well could thereby be detected by tetrazolium reduction, yielding a purple color. A pure *F. defendens* culture was prepared by centrifuging a starter culture for 5 min at 4000 rpm, after which the pellet was resuspended in the provided buffer (Biolog, USA). Then, 100 µl of the resultant pure *F. defendens* suspension (2.9x10^7^ CFU/mL) was added per well, and plates were sealed with parafilm and incubated for 48 hours at 28^°^C. The assay was conducted in triplicate, with *F. defendens* phenotypic fingerprints being established based upon the composition of the growth medium in wells in which a color change was visible at the end of the 48 hour culture period.

### Growth media preparation and growth conditions

To determine whether *F. defendens* inhibitory efficacy was influenced by culture conditions, *F. defendens* growth was assessed in four different types of medium (see Table S) under identical incubation conditions: (1) S-medium;^5,9^ (2) nutrient broth; (3) LB miller or Luria; (4) K9 minimal medium containing one of the following carbon source, 5 g/L of either glucose, galactose, or myoinositol, with or without 50 g/L of peptone (Sigma, USA) as a nitrogen source.

### Growth curves

*F. defendens* growth curves were established by adding 5 µl of bacterial culture starter cultures to each well of 96-well polystyrene microplates (Corning, USA) containing 200 µl of the tested medium in five replicates. Plates were then sealed with parafilm and incubated for 10 days at 28^°^C. Optical density at 595 nm (OD_595_) was measured daily using a EZ Read 400 plate reader (Biochrom Ltd., UK). Live cell counts (CFU/ml) were determined at 0, 4, and 10 days via 10-fold serial dilution and plating on 0.1 ml of Nutrient Agar, with the final count being established by multiplying the number of colonies by the corresponding dilution and volume factors. Growth rates were calculated as follows:

$$K=\left[\left(logN2-logN1\right)2.303\right]/\Delta t$$

In the above formula, K corresponds to the growth rate, N1 corresponds to the total bacterial count at the first time point, N2 corresponds to the total bacteria count at the second time point, and Δt corresponds to the difference between the two time points. Both time points were selected from the logarithmic phase of the growth curve.

For inhibition experiments, 20 ml of each designated medium was added to a 100 ml Erlenmeyer flask and mixed with 900 µl of bacterial culture starter grown in the same media, followed by incubation at 28°C with constant shaking at 120 rpm. After 4 and 10 days, filtrate samples were collected from each Erlenmeyer flask for downstream use in inhibition assays. These experiments were conducted using five replicate samples per condition in three independent experiments.

### *Assessment of filtrate-mediated inhibition of* S. melliferum growth

The inhibitory effects of the secreted metabolites prepared from *F. defendens* were assessed *in vitro* using the cultivatable Mollicutes *S. melliferum* as a phytoplasma surrogate, as detailed in previous studies.^5^ Briefly, 0.5 ml of filtrate was mixed with 0.5 ml of fresh S-medium in a 1.5 ml Eppendorf tube to which 10 μl of *S. melliferum* (~8x10^5^ cells) was added, followed by incubation for 5 days at 28°C. As a positive control, *S. melliferum* was cultured in fresh S-medium, while cell-free S-medium served as a negative control. Each treatment was conducted in triplicate. Inhibitory effects were assessed by adding 5 µl from each tube to 200 µl of S-medium containing phenol red in a polystyrene 96-well microplate, followed by incubation for 3–5 days. The OD_595_ values for each well were then measured as a correlate for cell growth, with higher (red) and lower (yellow) values corresponding to an absence of growth and growth, respectively. The minimum inhibitory concentration (MIC) of prepared filtrates was assessed by preparing serial two-fold filtrate dilutions in polystyrene 96-well microplates and testing the inhibitory activity for each well as detailed above.

### *Assessment of* F. defendens *crude filtrate effects on phytoplasma-infected periwinkle plantlet growth*

To test the *in planta* inhibitory effects of the prepared filtrates, we utilized the crude filtrate prepared in S-medium given that it was the only filtrate to exhibit *in vitro* inhibitory activity. The effects of the secreted metabolites contained within this filtrate on the growth of periwinkle plantlets infected with alfalfa witch’s broom phytoplasma were assessed. Plantlets were grown under controlled conditions (25°C, 14 h/10 h, day/night photoperiod) for 2 months in plant agar (MS media [Sigma M5524] supplemented with vitamins and solidified with 0.65% [w/v] agar) prior to experimental use. Plantlet roots were separated dipped in 1 mL of *F. defendens* filtrate or sterile S-medium (as a control) in a covered tube for 24 hours under sterile conditions. Each plantlet was then replanted in a glass tube containing 20 ml of fresh medium and grown under controlled conditions for 21 days. In total, nine replicates were included per treatment, and the growth and viability of these plants were assessed every seven days. At the end of this 21-day period, plantlets were removed from these tubes, and their biomass was measured.

### *Separation of* F. defendens *cells from secreted metabolites*

Filtration and small bioreactor platform (SBP)-based approaches were tested to separate *F. defendens* cells from secreted metabolites. For the filtration-based separation strategy, cells were cultured in a flask for 10 days, after which cell suspensions were centrifuged for 10 min at 5000 rpm at room temperature. Supernatants were then filtered through a 0.22 µm filter (FPV, JET BIOFIL, Spain). Two rounds of centrifugation and filtration were performed to obtain 20 ml of clear filtrate.

For the SBP approach, SBP capsules with semi-permeable membranes that retain microorganisms within the capsule while allowing their secreted metabolites to pass through were utilized. *F. defendens* cultures were grown within sealed SBP capsules (AC-20, BioCastle Water Technologies, Israel) by injecting 1 ml of *F. defendens* starter culture in S-medium into the capsule using a 25-G needle. Capsules were then sealed three times with a sealant containing cellulose acetate solution (8% cellulose acetate dissolved in Acetone, 88% and Methanol, 12% solution). and dried for ten minutes at room temperature according to provided instructions. Three different methods were then tested to achieve SBP capsule surface sterilization, as described below. After sterilization, each SBP capsule was submerged in 20 ml of sterile S-medium in a 50 ml tube and grown on a shaker at 120 rpm for 10 days at 28°C. Cell counts per SBP capsule were determined after removing the capsule from the growth medium and extracting the cultured bacteria from within using a syringe. The culture was then subjected to 10-fold serial dilutions, with 0.1 ml of the 10^−6^-10^−3^ dilutions then being plated on Nutrient Agar. Filtrates containing secreted metabolites were collected by removing the SBP with sterilized tweezers.

These experiments were conducted using five replicate samples per condition in three independent experiments, with the inhibitory effects of prepared filtrates on *S. melliferum* growth being assessed as detailed above.

### SBP capsule surface sterilization

Three different techniques were tested to establish the optimal approach to SBP capsule surface sterilization. Briefly, capsules containing *F. defendens* cells were (1) dipped in 70% ethanol and then rinsed with sterile saline; (2) irradiated for 30 minutes per side with an ultraviolet (UV) illuminator; or (3) dipped in 70% ethanol and then subjected to UV irradiation (30 min/side). After sterilization, individual capsules were placed in 20 ml of S-medium in a 50 ml tube and incubated for 10 days, with medium clarity being assessed on a daily basis. Tubes were considered to be contaminated if the media therein turned opaque during the observation period. When these capsules were surface sterilized with 70% ethanol or UV radiation, 50% and 75% of the tubes containing *F. defendens*-SBP were contaminated, respectively. The most effective surface sterilization method was immersing the SBP capsule in 70% ethanol, followed by UV irradiation for 30 min. When this method was used, no contamination of SBP (0%) was observed.

### Statistical analysis

Descriptive statistics (i.e., means with standard error) were calculated using Microsoft Excel. To compare growth rates of *F. defendens* and levels of inhibitory activity, one-way analyses of variance (ANOVAs) were performed using SPSS (IBM, USA).

## Results and discussion

### F. defendens *biochemical characterization*

Initially, the growth requirements of *F. defendens* were determined by modulating multiple variables using a GEN MicroPlate test kit (Figure S1). Following a two-day incubation period, this analysis revealed that *F. defendens* was able to utilize D-galactose, D-glucose, myoinositol, D-fucose, and N-acetyl-glucosamine. These bacteria were unable to grow at a pH below 6 or at NaCl concentrations above 1% (Figure S1). *F. defendens* also appeared to be resistant to lincomycin, vancomycin, troleandomycin, and rifamycin.

### *Assessment of* F. defendens *growth in different media*

**Figure 1.**
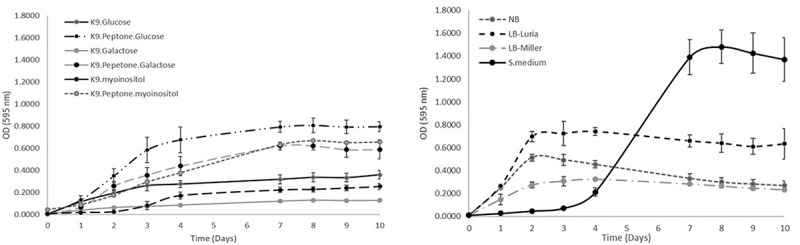
*F. defendens* bacterial growth curves in different media as was measured by total count using spectrophotometer. A. Defined media based on K9 minimal salt medium, supplemented with a carbon source (glucose, galactose, or myoinositol), with or without peptone. B. Rich non-defined media: S-medium, NB, LB (LB Miller and LB Luria). The results were calculated from five replicates samples assessed for each medium formulation in three repeated experiments.

In light of the above results, we prepared different formulations of growth media in an effort to optimize *F. defendens* growth and metabolite secretion. Specifically, two types of media were assessed – defined salt media and rich, non-defined media. For defined salt media, a variation on classic M9 media was used in which salts were K^+^-based rather than Na^+^-based (K9 media) owing to the observed sensitivity of *F. defendens* to NaCl. The growth of *F. defendens* in K9 media supplemented with various sugars as the sole carbon source (galactose, glucose, or myoinositol) with or without peptone supplementation was assessed. The tested rich, non-defined media types included S-medium, NB, LB-Miller, and LB-Luria media. Distinct patterns of *F. defendens* growth, as measured based on absorbance, were observed for each tested media type (Figure 1). The primary difference observed among media types was the time required to reach the stationary phase, which was reached in all K9-based media other than K9+ mioinositol within 7 days (Figure 1a), while cultures grown in LB, NB, and K9+ mioinositol reached the stationary phase after just three days (Figure 1b). When grown in minimal medium containing myoinositol, *F. defendens* cultures exhibited a two-day lag phase, followed by a two-day logarithmic growth phase, after which they reached stationary phase. Overall, lower growth rates were observed on average when cells were cultured in either minimal medium or rich media containing high Na^+^ concentrations. The growth rate of *F. defendens* was relatively low (0.023–0.046 1/h) compared to other biocontrol agents such as *Bacillus subtilis R14*^26^ and *Pseudomonas* strain HHRE81,^27^ which showed a higher growth rate of 1.2 1/h and 0.76 1/h, respectively. Nevertheless, the maximal cell concentrations of these bBCAs did not exceed 10^9^ CFU/ml in all media examined, as was demonstrated in *F. defendens* in the current study (Table 1).

**Table 1.** The effects of different growth media on *F. defendens* total cell counts (OD_595nm_) and live cell counts (CFU/ml)

Media/day	0		4		10		Growth rate K ± S.D (1/hr)*
	OD_595nm_	CFU/ml	OD_595nm_	CFU/ml	OD_595nm_	CFU/ml	
NB	0.010	1.06x10^7^	0.453	7.3x10^8^	0.296	1.91x10^9^	0.040 ± 0.009 a**
LB Luria	0.006	5.0x10^6^	0.325	1.1x10^9^	0.232	5.0x10^8^	0.036 ± 0.001 a
LB Miller	0.007	5.6x10^6^	0.742	1.04x10^9^	0.633	1.2x10^8^	0.023 ± 0.004 b
S-medium	0.010	7.7x10^6^	0.212	1.0x10^7^	1.370	1.0x10^7^	0.035 ± 0.018 a
K9-Glucose	0.007	1.3x10^7^	0.276	1.90x10^8^	0.360	3.5x10^8^	0.024 ± 0.004 b
K9- Glucose Peptone	0.007	1.33x10^7^	0.677	ND	0.797	1.20x10^9^	0.033 ± 0.006 a
K9-Galactose	0.005	1.44x10^7^	0.086	1.6x10^8^	0.128	1.7x10^8^	0.030 ± 0.006 ab
K9- Galactose Peptone	0.009	1.47x10^7^	0.439	9.0x10^6^	0.589	1.04x10^9^	0.046 ± 0.001 c
K9-myoinositol	0.008	8.0x10^6^	0.172	3.0x10^6^	0.254	7.0x10^6^	0.034 ± 0.02 ab
K9- myoinositol Peptone	0.045	5.0x10^6^	0.379	3.0x10^7^	0.658	ND	0.027 ± 0.003 b

* data calculated from OD_595nm_ at log phase; ** Means denoted by a different letter indicate significant differences between treatments (p < 0.05); ND- not detected.

The above results suggest that *F. defendens* is a prototrophic bacterium capable of growing in minimal media containing different types of sugars included as the sole carbon source. However, as with many other bacteria, its growth was better in complex media containing amino acids and other nutrients as evidenced by the significant improvements in *F. defendens* growth in K9 medium containing glucose and galactose when media was supplemented with peptone (Table 1). Interestingly, *F. defendens* growth in the S-medium (S) exhibited a unique pattern characterized by a late and abrupt increase in OD_595_ during the log phase, whereas the population size (measured in CFU/mL) remained constant from days 4–10 at ~1x10^7^ CFU/mL (Table 1). This suggests that the increase in OD_595_ was attributable to a change in media color, which turned brown, rather than a result of an increase in the number of live bacterial cells. We speculate that this darker media coloration was the result of secreted and/or degraded metabolites released by *F. defendens.*

### *Assessment of the inhibitory activity of* F. defendens *filtrates on* S. melliferum *growth*

**Figure 2.**
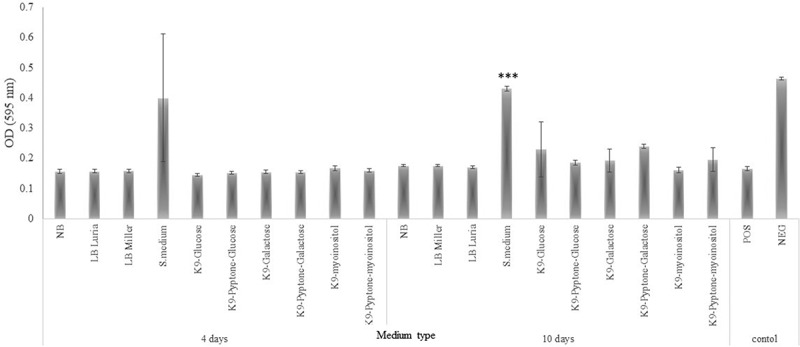
The inhibitory effect of different *F. defendens* filtrates on *S. melliferum* growth in culture at two time points. Filtrates were tested after 4 and 10 days of *F. defendens* growth prior to filtration. Higher OD values correspond to lower growth. Sterile medium served as a negative control (NEG), and *S. melliferum* (approx. 1x10^7^CFU/ml) was cultured in S-medium as a positive control (POS). Data are given as means with standard deviations from triplicate samples, with results being representative of one out of three biological experiments. ***P < .01.

Cultures of the phytoplasma surrogate *S. melliferum* were next grown in the presence *F. defendens* filtrates prepared in the different media formulations discussed above. Only the filtrate prepared from *F. defendens* grown in S-medium significantly inhibited *S. melliferum* growth (Figure 2). The fact that only a very rich growth medium enabled the secretion of metabolites that inhibit cell growth of *S. melliferum* implies that these secondary metabolites are produced under stress conditions that were generated in this case by the high osmotic pressure caused by a high sugar dose. Moreover, growing the cells in other rich media did not result in the secretion of inhibitory metabolites, suggesting that *F. defendens* need unique source materials for their production. These results are consistent with prior evidence that growth medium can influence bBCA antibiotic activity, with *Pseudomonas fluorescens* strains AS15 and UTPF61 exhibiting distinct patterns of growth, metabolite secretion, and inhibitory activity against *Rhizoctonia solani* and *Sclerotinia sclerotiorum*, respectively, when grown in different types of media.^25,28^

The inhibitory activity of *F. defendens* filtrates was increased following a 10-day culture period (Figure 2), suggesting that inhibitory metabolite secretion likely occurs primarily during the stationary phase and reinforcing the hypothesis that these inhibitory substances are probably secondary metabolites, produced under stress conditions. Similarly, secondary metabolites of the endophytic bacterial strains *Bacillus megaterium NBRC* and *Pseudomonas protegens* MP12 inhibit the growth of phytopathogenic bacteria and phytopathogenic fungi, respectively.^29,30^

### In planta *examination of the inhibitory activity of* F. defendens *filtrates*

**Figure 3.**
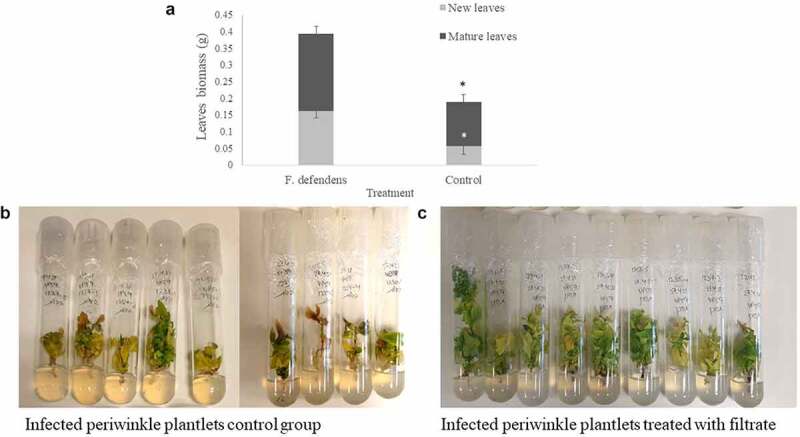
The effects of *F. defendens* filtrate treatment on the growth of phytoplasma-infected periwinkle plantlets at 21 days post-treatment. A. Plant biomass of mature and new leaves. B. Control: infected periwinkle plantlets treated with 1 mL of filtrate prepared from sterile S-medium. C. infected periwinkle plantlets treated with 1 mL of filtrate prepared from a 10 day *F. defendens* cell suspension. Each plantlet was dipped in filtrate for 24 h before being replanted in plant medium. Data are given as means with standard deviations from nine replicant samples. *P < .05.

To test the *in planta* relevance of the above *in vitro* data, the roots of phytoplasma-infected periwinkle plants were dipped in filtrates collected from *F. defendens* after culture for 10 days in S-medium. Following a 21-day growth period, the leaf biomass of treated plantlets was doubled compared to control plantlets (Figure 3a). This difference was the result of the growth of both mature and newly emergent leaves (Figure 3a), suggesting that, following filtrate treatment, both leaf developmental stages exhibited decreased symptoms of yellows disease. In general, a higher number of leaves were observed in treated plantlets, and the leaves were greener and more prominent, while in the control group, plantlets were characterized by fewer leaves, many of which had turned yellow-brown (Figure 3b,c). These results emphasize the beneficial effect of the secreted metabolites derived from *F. defendens* on phytoplasma-infected plants, consistent with previously published results.^7^

### *SBP capsules enable the effective separation of* F. defendens-*derived metabolites*

Given that *F. defendens* cells are relatively small, separating them from secreted substances is challenging as not all bacteria can be readily pelleted during centrifugation, resulting in the filtration of the collected supernatants being slow and often leading to the clogging of the filter. As such, we sought to resolve this problem by enabling *F. defendens* cells to grow within a separate, spatially confined area such that their secreted metabolites could be rapidly and reliably collected. Consequently, we tested the use of the Small Bioreactor Platform (SBP; AC-20, BioCastle Water Technologies, Israel) under the hypothesis that *F. defendens* would be able to grow within SBP capsules, while the metabolites secreted from these cells would be able to pass into the sterile growth media in which the SBP was submerged.

SBP capsules have previously been evaluated for their ability to encapsulate bacteria including *Pseudomonas putida*,^31^ *Rhodococcus zopfii*,^32^ and *Delftia* EROSY^33^ in an effort to achieve the efficient degradation of a range of pollutants by maintaining high bacterial concentrations and viability within these capsules. However, this report is the first to our knowledge in which SBP capsules have been evaluated under sterile conditions as a tool for readily and rapidly separating cells from their secreted metabolites.

Following the 10-day incubation period, the external media surrounding SBP capsules remained cell-free, while the number of viable *F. defendens* cells present in suspended media (CFU/mL) was similar to the number present within SBP capsules (1.01x10^8^ ± 4x10^6^ and 2.06 × 10^8^ ± 1.3x10^7^, respectively). These results confirmed that bacterial viability was maintained within the SBP. In addition, the MIC of the filtrate prepared from these SBP capsules was at a dilution of 25%, which was similar to the MIC of filtrate prepared via the conventional method, suggesting that SBP capsules do not limit the secretion and/or diffusion of inhibitory metabolites. SBP membrane pore size is approximately 0.2 µm, while the cell size of *F. defendens* is 1–1.3 µm.^34^ Therefore, it was assumed that the bacterium cell is larger than the capsule pore size and cannot pass through the membrane into the growth medium. The results confirmed that capsule pores are large enough to enable the trafficking of metabolites with inhibitory activity across the membrane into the external filtrate.

## Conclusions

In summary, the results of this study indicate that *F. defendens* requires very rich media with specific characteristics in order to secrete high concentrations of secondary metabolites capable of inhibiting *S. melliferum* growth as well as reducing the symptoms of phytoplasma infection in plantlets. Moreover, we found that SBP capsules were able to effectively confine *F. defendens* cells but not their secreted secondary metabolites, which consequently could be easily collected. This method provides an efficient and reliable approach to the preparation of filtrates with inhibitory activity.

We suggest further examining the role of each component of S-medium in the cell growth and metabolite secretion of *F. defendens* in order to discover the factors that allow Mollicutes inhibition by this beneficial bacterium.

## References

[1] Lee IM, Davis RE, Gundersen-Rindal DE. Phytoplasma: phytopathogenic mollicutes. Ann Rev Microbiol. 2000;54:221–8. doi:10.1146/annurev.micro.54.1.221. 11018129

[2] Weintraub PG, Wilson MR, Jones P. Control of phytoplasma diseases and vectors. Phytoplasmas, 13. 2010;233–249.

[3] Gonella E, Musetti R, Crotti E, Martini M, Casati P, Zchori-Fein E. Microbe relationships with phytoplasmas in plants and insects. In Bertaccini, A., Weintraub, P., Rao, G., Mori, N (Eds.) Phytoplasmas: plant pathogenic bacteria-II 2019. Singapore: Springer; 207–235.

[4] Latha P, Karthikeyan M, Rajeswari E. Endophytic bacteria: prospects and applications for the plant disease management. In Ali Ansari, R., Mahmood, I (Eds.). Plant health under biotic stress 2019. Singapore: Springer; 1–50.

[5] Iasur-Kruh L, Naor V, Zahavi T, Ballinger MJ, Sharon R, Robinson WE, Perlman SJ, Zchori-Fein E. Bacterial associates of *Hyalesthes obsoletus* (Hemiptera: cixiidae), the insect vector of bois noir disease, with a focus on cultivable bacteria. Res Microbiol. 2017;168:94–101. doi:10.1016/j.resmic.2016.08.005. 27602526

[6] Lidor O, Dror O, Hamershlak D, Shoshana N, Belausov E, Zahavi T, Mozes‐Daube N, Naor V, Zchori‐Fein E, Iasur‐Kruh L, et al. Introduction of a putative biocontrol agent into a range of phytoplasma‐and liberibacter‐susceptible crop plants. Pest Manag Sci. 2018;74:811–819. doi:10.1002/ps.4775. 29072824

[7] Iasur-Kruh L, Zahavi T, Barkai R, Freilich S, Zchori-Fein E, Naor V. *Dyella*-like bacterium isolated from an insect as a potential biocontrol agent against grapevine yellows. Phytopathology. 2018;108:336–341. doi:10.1094/PHYTO-06-17-0199-R. 28990480

[8] Lahav T, Zchori-Fein E, Naor V, Freilich S, Iasur-Kruh L. Draft genome sequence of a Dyella-like bacterium from the planthopper Hyalesthes obsoletus. Genome Announc. 2016 Jul 21;4(4):e00686–16. doi:10.1128/genomeA.00686-16. 27445378 PMC4956451

[9] Naama-Amar A, Gitman S, Shoshana N, Bahar O, Naor V, Zchori-Fein E, Iasur-Kruh L. Antimicrobial activity of metabolites secreted by the endophytic bacterium *Frateuria defendens*. Plants. 2020;9:72. doi:10.3390/plants9010072. PMC7020481 31935875

[10] Naor V, Zahavi T, Barkai R, Weiss N, Mozes-Daube N, Dror O, Finkelstein C, Ahron S, Bahar O, Zchori-Fein E, et al. *Frateuria defendens* reduces yellows disease symptoms in grapevine under field conditions. Vitis. 2021;60:109–117.

[11] Stanbury PF, Whitaker A, Hall SJ. The recovery and purification of fermentation products. In: Stanbury PF, Whitaker A, Hall SJ, editors. Principles of Fermentation Technologym. 2017. p. 619–686. Elsevier.

[12] Oyewole O, Kareem S, Adeleye T. Biotechnologies/fermentation technologies for large-scale industrial enzyme production for the food and beverage industry. Ferment Algal Biotechnologies Food Beverage Other Bioprod Ind. 2022;3:41–67.

[13] Hutchinson UF, Gqozo S, Jolly NP, Chidi BS, Du Plessis HW, Mewa-Ngongang M, Ntwampe SK. Aeration, agitation and cell immobilization on corncobs and oak wood chips effects on balsamic-styled vinegar production. Foods. 2019 Aug;8(8):303. doi:10.3390/foods8080303. PMC6723862 31374870

[14] Karagoz P, Bill RM, Ozkan M. Lignocellulosic ethanol production: evaluation of new approaches, cell immobilization and reactor configurations. Renewable Energy. 2019 Dec 1;143:741–752. 10.1016/j.renene.2019.05.045.

[15] Gao H, Lu J, Jiang Y, Fang Y, Tang Y, Yu Z, Zhang W, Xin F, Jiang M. Material‐mediated cell immobilization technology in the biological fermentation proces. Biofuels Bioprod Biorefin. 2021 Jul;15(4):1160–1173. doi:10.1002/bbb.2219.

[16] Li G, Liu J, Chen N, Xu Q. A new method to recover L-tyrosine from E. coli fermentation broth. Bioengineered. 2020 Jan 1;11(1):1080–1083. doi:10.1080/21655979.2020.1827893. 33094662 PMC8291781

[17] Dos Santos Nv, de Carvalho Santos‐ebinuma V, Pessoa Junior A, Pereira JF. Liquid–liquid extraction of biopharmaceuticals from fermented broth: trends and future prospects. J Chem Technol Biotechnol. 2018 Jul;93(7):1845–1863. doi:10.1002/jctb.5476.

[18] Sadare OO, Ejekwu O, Moshokoa MF, Jimoh MO, Daramola MO. Membrane purification techniques for recovery of succinic acid obtained from fermentation broth during bioconversion of lignocellulosic biomass. Curr Adv Future Perspect Sustainability. 2021 Jan;13(12):6794.

[19] Zhang QW, Lin LG, Ye WC. Techniques for extraction and isolation of natural products: a comprehensive review. Chin Med. 2018 Dec;13(1):1–26. doi:10.1186/s13020-018-0177-x. 29692864 PMC5905184

[20] Schuster A, Schmoll M. Biology and biotechnology of *Trichoderma* Appl Microbiol Biotechnol. 2010;87:787–799. doi:10.1007/s00253-010-2632-1. 20461510 PMC2886115

[21] De la Fuente M, Vidal JM, Miranda CD, González G, Urrutia H. Inhibition of *Flavobacterium psychrophilum* biofilm formation using a biofilm of the antagonist *Pseudomonas fluorescen* s FF48. SpringerPlus. 2013;2:1–9. doi:10.1186/2193-1801-2-176. 23667820 PMC3650236

[22] Shi L, Liang Z, Li J, Hao J, Xu Y, Huang K, Tian J, He X, Xu W. Ochratoxin A biocontrol and biodegradation by *Bacillus subtilis* CW14. J Sci Food Agric. 2014;94:1879–1885. doi:10.1002/jsfa.6507. 24293396

[23] Vinale F, Nicoletti R, Borrelli F, Mangoni A, Parisi OA, Marra R, Lombardi N, Lacatena F, Grauso L, Finizio S, et al. Co-culture of plant beneficial microbes as source of bioactive metabolites. Sci Rep. 2017;7:1–2. doi:10.1038/s41598-017-14569-5. 29085019 PMC5662714

[24] Rana A, Sahgal M, Kumar P. Biocontrol Prospects of *Pseudomonas fluorescens* AS15 against banded leaf and sheath blight disease of maize under field condition in conducive soil. National Acad Sci Lett. 2019;42:425–428. doi:10.1007/s40009-018-0772-5.

[25] Rana A, Sahgal M. Evaluation of bio control efficacy *of Pseudomonas fluorescens* AS15 against banded leaf and sheath blight disease pathogen (*Rhizoctonia solani*) in different carbon and nitrogen sources. Int J Curr Microbiol Appl Sci. 2017;6:1347–1353. doi:10.20546/ijcmas.2017.606.158.

[26] Luna CL, Mariano RL, Souto-Maior AM. Production of a biocontrol agent for crucifers black rot disease. Braz J Chem Eng. 2002 Apr;19(2):133–140. doi:10.1590/S0104-66322002000200007.

[27] Srivastava R, Aragno M, Sharma AK. Cow dung extract: a medium for the growth of *Pseudomonads enhancing* their efficiency as biofertilizer and biocontrol agent in rice. Indian J Microbiol. 2010 Sep;50(3):349–354. doi:10.1007/s12088-010-0032-y. 23100852 PMC3450066

[28] Heidari-Tajabadi F, Ahmadzadeh M, Moinzadeh A, Khezri M. Influence of some culture media on antifungal activity of *Pseudomonas fluorescens* UTPF61 against the *Sclerotinia sclerotiorum*. Afr J Agric Res. 2011;6:6340–6347.

[29] Liu JM, Wang SS, Zheng X, Jin N, Lu J, Huang YT, Fan B, Wang FZ. Antimicrobial activity against phytopathogens and inhibitory activity on solanine in potatoes of the endophytic bacteria isolated from potato tubers. Front Microbiol. 2020;11. 10.3389/fmicb.2020.570926 PMC7705204 33281766

[30] Andreolli M, Zapparoli G, Angelini E, Lucchetta G, Lampis S, Vallini G. *Pseudomonas protegens* MP12: a plant growth-promoting endophytic bacterium with broad-spectrum antifungal activity against grapevine phytopathogens. Microbiol Res. 2019;1:123–131. doi:10.1016/j.micres.2018.11.003. 30642463

[31] Kurzbaum E, Raizner Y, Cohen O, Suckeveriene RY, Kulikov A, Hakimi B, Kruh LI, Armon R, Farber Y, Menashe O. Encapsulated *Pseudomonas putida* for phenol biodegradation: use of a structural membrane for construction of a well-organized confined particle. Water Res. 2017;121:37–45. doi:10.1016/j.watres.2017.04.079. 28505532

[32] Menashe O, Raizner Y, Kuc ME, Cohen-Yaniv V, Kaplan A, Mamane H, Avisar D, Kurzbaum E. Biodegradation of the endocrine-disrupting chemical 17α-ethynylestradiol (EE2) by *Rhodococcus zopfii* and Pseudomonas putida encapsulated in small bioreactor platform (SBP) capsules. Appl Sci. 2020;10:336. doi:10.3390/app10010336.

[33] Oz YB, Mamane H, Menashe O, Cohen-Yaniv V, Kumar R, Kruh LI, Kurzbaum E. Treatment of olive mill wastewater using ozonation followed by an encapsulated acclimated biomass. J Environ Chem Eng. 2018;6:5014–5023. doi:10.1016/j.jece.2018.07.003.

[34] Lidor O, Santos-Garcia D, Mozes-Daube N, Naor V, Cohen E, Iasur-Kruh L, Bahar O, Zchori-Fein E. Frateuria defendens sp. nov., bacterium isolated from the yellows grapevine’s disease vector Hyalesthes obsoletus. Int J Syst Evol Microbiol. 2019 May 1;69(5):1281–1287. doi:10.1099/ijsem.0.003305. 30785390

